# Effect of Ultrasonic Surface Mechanical Attrition Treatment-Induced Nanograins on the Mechanical Properties and Biocompatibility of Pure Titanium

**DOI:** 10.3390/ma15155097

**Published:** 2022-07-22

**Authors:** Furqan Ahmed, Muhammad Zain-ul-abdein, Iftikhar Ahmed Channa, Muhammad Kamran Yaseen, Sadaf Jamal Gilani, Muhammad Atif Makhdoom, Muhammad Mansoor, Usman Shahzad, May Nasser bin Jumah

**Affiliations:** 1 Department of Metallurgical and Materials Engineering (MME), University of Engineering and Technology (UET), Lahore 54890, Pakistan; furqan.ahmed@uet.edu.pk (F.A.); kamran.met@gmail.com (M.K.Y.); 2 Thin Film Lab, Department of Metallurgical Engineering, NED University of Engineering & Technology, Karachi 75270, Pakistan; iftikharc@neduet.edu.pk; 3 Department of Basic Health Sciences, Preparatory Year, Princess Nourah Bint Abdulrahman University, Riyadh 11671, Saudi Arabia; sjglani@pnu.edu.sa; 4 Institute of Metallurgy and Materials, University of the Punjab, Lahore 54590, Pakistan; atif.imme@pu.edu.pk; 5 Institute of Industrial Control Systems, Rawalpindi 46000, Pakistan; malik01677@yahoo.com; 6 Materials Engineering Division, Consulting Services Department, Saudi Aramco, Dhahran 31311, Saudi Arabia; ushahzad@hotmail.com; 7 Biology Department, College of Science, Princess Nourah Bint Abdulrahman University, Riyadh 11671, Saudi Arabia; mnbinjumah@pnu.edu.sa; 8 Environment and Biomaterial Unit, Health Sciences Research Center, Princess Nourah Bint Abdulrahman University, Riyadh 11671, Saudi Arabia; 9 Saudi Society for Applied Science, Princess Nourah Bint Abdulrahman University, Riyadh 11671, Saudi Arabia

**Keywords:** biocompatibility, surface mechanical attrition treatment, titanium, nanograins, mechanical properties

## Abstract

Commercially pure titanium (Ti) is widely used in bio-implants due to its high corrosion resistance. However, Ti exhibits marginally low mechanical and tribological properties, which limit its applications in some orthopedic implants. In this work, the Ti samples were subjected to ultrasonic surface mechanical attrition treatment (SMAT) for various durations to improve their surface properties such as hardness, strength and surface energy. SMAT-induced grain refinement was analyzed using optical, scanning electron and atomic force microscopy techniques. A Vickers hardness test was performed to determine the through-thickness hardness. Mechanical testing was carried out to measure the yield strength, ultimate tensile strength and ductility of the specimens. Corrosion tests were performed on a Gamry Potentiostat. The surface energy of SMAT-modified samples was calculated using the Owens–Wendt method. It was observed that SMAT reduced the average grain size from 50 μm to as low as 100 nm. The grain refinement and the corresponding grain boundary density led to a significant improvement in mechanical properties and biocompatibility in terms of increased hardness, yield and tensile strengths, surface energy, corrosion rate and hydrophilicity.

## 1. Introduction

Recent trends in biomedical applications have led to the usage of a large variety of materials that must exhibit specific physical and mechanical properties, including biocompatibility [1,2]. The properties of the materials used in the body implants should be comparable with ‘autogenous tissues’ and should not have any harmful effect on the body. Biocompatibility, bio-functionality, bio-adhesion and corrosion resistance are the main characteristics that determine the suitability of any material to act as an implant [3]. Titanium (Ti) has been one of the most frequently used implant materials for decades due to its good mechanical properties, excellent corrosion resistance and superior biocompatibility [4]. Many authors have shown that Ti has outstanding in-vivo and in-vitro compatibility [5,6], thanks to its low electrical conductivity resulting from the formation of a thin passive oxide film at the surface of the implant [7]. The oxide film, in addition, restricts the implant’s corrosion and maintains physiological pH value [8] since it has an isoelectric point of 5–6 [1]. In an aqueous environment, Ti has low reactivity with macromolecules and less tendency for ion formation [9]. 

The implants made of pure Ti and the alloy Ti6Al4V are mostly used on a commercial scale. The presence of vanadium (V) in Ti_6_Al_4_V, however, is a cause of cytotoxicity according to some studies [10,11]. Moreover, the modulus of elasticity of Ti_6_Al_4_V is much higher than that of cortical bone, which is undesirable [12,13]. Hence, given its superior biocompatible attributes, almost all permucosal dental implants are manufactured with commercially pure Ti [14]. Furthermore, it is desirable that a prosthetic implant must possess enough mechanical strength so that its load-bearing capacity is at least three to four times the body weight. This includes the resistance to cyclic loading as some implants experience up to 5 million cycles per year [15]. Although several metallic materials offer good fatigue resistance, corrosion remains one of the major causes of mechanical failure of implants [16]. Overall, commercially pure Ti is used in bio-implants in four different grades, where each grade has a different set of mechanical properties and its use is also application-specific [17,18]. Their strengths, generally, range from 240 MPa to 550 MPa [1,19]. An insufficient strength level, however, limits their applications in many orthopedic endoprostheses [20].

Over the past few years, progress in the field of nanotechnology has modernized the research and development of bio-implants. To produce nanosized grains or phases in metals and alloys, severe plastic deformation (SPD) techniques are effective choices as they have high reproducibility. Surface nano-crystallization (SNC) is an efficient way to selectively improve the tribological characteristics of the surface of metals or alloys without altering the properties of the core [21,22]. Surface mechanical attrition treatment (SMAT) is an SPD technique in which SNC is achieved at the surface of the bulk material. Recently, several researchers have reported improvement in wear resistance and surface hardness by achieving SNC through SMAT [23,24,25,26]. It has been identified that a high grain boundary density promotes a stable passive film. The nanograins produced by SMAT increase the surface grain boundary density, which, in turn, increases Ti’s tendency to form a stable oxide film [27]. The native passive oxide layer formed at the surface of the coarse-grained Ti is not bioactive enough to support a direct bond with surrounding bones. Such Ti implants are prone to forming fibrous tissues at the implant-bone interface, which may cause a lack of osseointegration and enhance the possibility of implant joint loosening with the passage of time [28,29]. In many failure cases due to low surface hardness, the implants form wear debris, which not only causes infection but also necessitates the replacement of the artificial joint. However, an increase in hydrophilicity by the deposition of non-toxic salts on the roughened surface of implants, however, has shown promise in improving osseointegration [30].

In the present work, the improvement in mechanical properties and biocompatibility of commercially pure Ti by ultrasonic SMAT-induced surface nanograins has been investigated. Note that the property of hydrophilicity was used as a measure of biocompatibility.

## 2. Materials and Methods

### 2.1. Ultrasonic Surface Mechanical Attrition Treatment

Polished samples of the so-called Grade-4 commercially pure Ti, including Fe, Si, O, N, H, C and Al with wt.% of 0.10, 0.01, 0.16, 0.014, 0.004, 0.022 and 0.23, respectively, were subjected to ultrasonic SMAT for 15, 20 and 25 min using 30 g steel balls of diameter 3 mm. The distance between samples and the ultrasonic base plate was maintained at 12 mm. Ultrasonic generator frequency was kept at 20 kHz, which was employed for all sets of parameters. The treated samples were then cleaned with 5% HCl solution followed by rinsing in water to get rid of adherent steel content at the surface of the samples resulting from the repetitive impacts of steel balls on the samples.

### 2.2. Microscopy and Hardness Testing

The cleaned samples were then sectioned using a diamond cutter for characterization. Surface morphology of the samples was observed using a scanning electron microscope (SEM); model: JEOL JSM-5910LV, 2000 (JEOL Technics Ltd., Akishima, Japan). The SEM surface characterization was performed using a secondary electron detector at an acceleration voltage of 20 kV while maintaining a pressure of 10^−5^ mbar. 

The cut sections of all treated samples were mounted in Bakelite using steel strips at their edges to avoid edge roundness. Polishing of the samples was performed with 0.03 µm alumina powder. The thicknesses of the SMAT-modified layers were determined using a displacement test and a micro-Vickers hardness tester (Model: LECO DM-400T, Saint Joseph, MI, USA) with a 25 g load applied at different locations from the surface of the samples to the center. These samples were, later on, etched with Kroll’s reagent to reveal microstructure. The grain size of untreated Ti sample was determined using optical microscope. The average grain size was measured using Olympus-Stream Motion software.

The etched samples of SMAT modified surfaces treated for 15, 20 and 25 min were then analyzed using Nanosurf Flex atomic force microscope (AFM), model: Nanosurf AG, Liestal, Switzerland 2012, equipped with Silicon AFM Probe (Top 190Al-G) in contact mode at a resonant frequency of 190 kHz with a force constant of 48 N/m. The samples of dimensions 5 mm × 10 mm × 10 mm each were electrically ground and mounted on a static stage before scanning. 

### 2.3. Surface Energy Calculation

The surface energy of the samples was determined by Owens–Wendt (OW) method using distilled water and ethylene glycol. For this purpose, an ASTM D7334 standard and the following equation were implemented:

(1) $$\left(1+cos\theta \right)\gamma_{L}=2\left(\sqrt{\gamma_{L}^{d}\times \gamma_{S}^{d}}+\sqrt{\gamma_{L}^{p}\times \gamma_{S}^{p}}\right)$$

where *θ* is a contact angle of liquid on the solid surface and γ is surface energy. The subscripts *L* and *S* refer to liquid and solid, respectively; while the superscripts d and p denote the dispersive and polar components, respectively. Surface energy of the solid ($\gamma_{S}$) will be a sum of $\gamma_{S}^{d}$ and $\gamma_{S}^{p}$, which are the dispersive and polar components of surface energies of solid, respectively. The values of surface energy of liquids, $\gamma_{L}$, and their corresponding components, $\gamma_{L}^{d}$ and $\gamma_{L}^{p}$, of distilled water and ethylene glycol are readily available in the literature [31] and are reproduced in Table 1.

### 2.4. Tensile Testing

**Figure 1:**
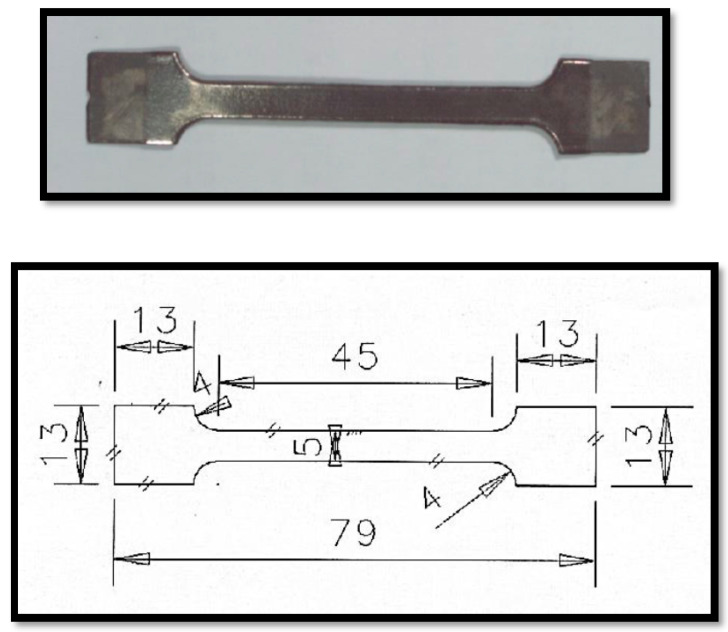
Tensile test specimen (all dimensions in mm).

Tensile tests of untreated and SMAT-modified samples were carried out on dog-bone type specimens according to the ASTM E8 standard using Universal Testing Machine (Model: INSTRON 1195, Instron Limited, High Wycombe, UK) at a displacement rate of 0.5 mm/min. The tensile test samples with a gauge length of 25 mm, width 5 mm and thickness 1 mm were prepared by modifying both the top and bottom surfaces through SMAT. The specimen geometry is shown in Figure 1.

### 2.5. Corrosion Testing

All the treated and untreated samples were subjected to corrosion testing, which was carried out on a Gamry Potentiostat, model: PCI/750 Potentiostat/ZRA, Gamry Instruments, Warminster, PA, USA. Stimulated body fluid (Ringer Lactate) was used as an electrolyte for potentiodynamic polarization. Tafel scan was performed using a saturated calomel electrode as a reference electrode and a graphite electrode as a counter electrode at 37 °C. A scan rate of 0.012 mV was applied during the test.

## 3. Results and Discussion

### 3.1. Surface Topography

**Figure 2:**
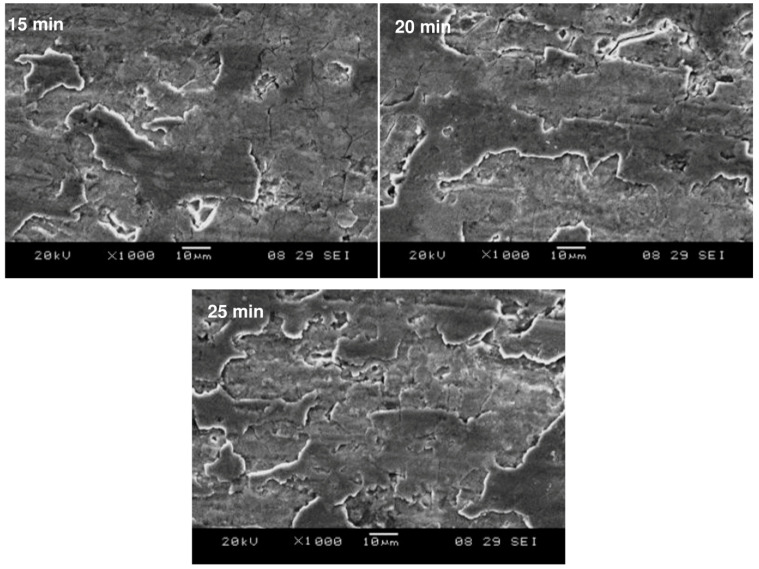
Surface topography of the SMAT modified samples treated for 15 min, 20 min and 25 min.

The surface topography of SMAT-modified samples treated for 15 min, 20 min and 25 min showed high surface roughness. Figure 2 highlights the presence of roughened patches on the surface of each sample observed through SEM. The average surface roughness values (R_A_) of the samples treated for 15 min, 20 min and 25 min were found to be 0.18 μm, 0.18 μm and 0.16 μm, respectively. Note that the initial surface roughness of all the specimens was ≤0.03 μm. Clearly, the increase in surface roughness is a consequence of the repetitive impacts of steel balls onto the polished sample surface. There is, however, a limit to this increase in roughness since the plastic deformation only involves impact loading through smooth spherical balls. For this very reason, there is no appreciable difference in the roughness values despite the increase in the duration of SMAT by a factor from 1.3 to 1.7. It may, therefore, be stated that the surface roughness reached its saturation within the first 15 min of SMAT.

### 3.2. Microstructural Analysis

**Figure 3:**
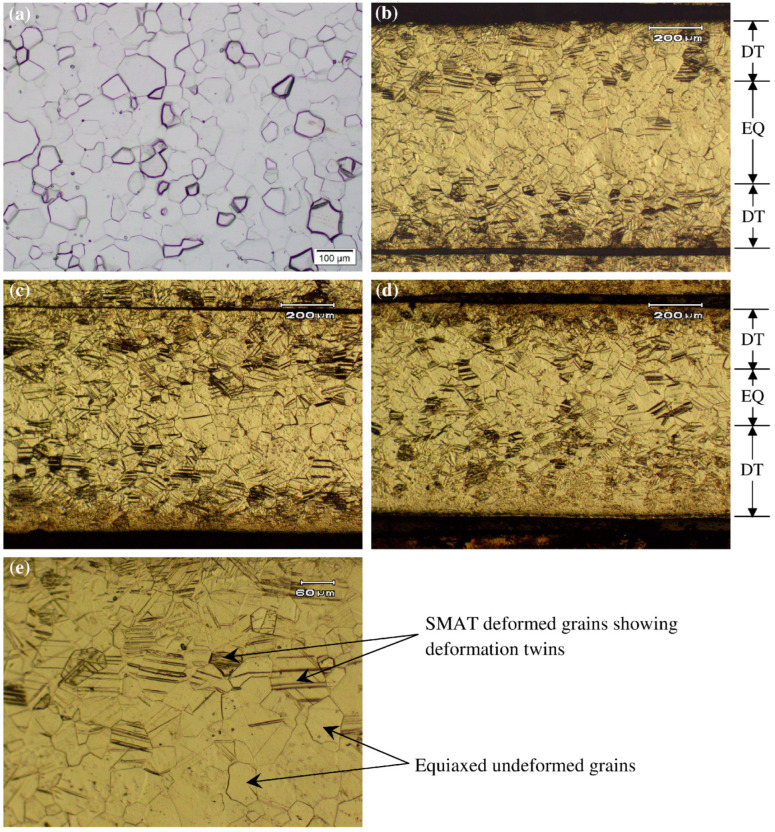
Optical microstructures of: (**a**) untreated Ti with equiaxed grains; SMAT-modified samples treated for: (**b**) 15 min; (**c**) 20 min and (**d**) 25 min showing twined structure at both surfaces and undeformed equiaxed grains at the center; (**e**) SMAT deformed and undeformed equiaxed grains at higher magnification. DT and EQ stand for deformation twins and equiaxed grains.

Optical microscopy provides evidence of SMAT-induced plastic deformation on the top and bottom surfaces of the samples. Figure 3 compares the microstructures of the cross-section of untreated Ti and SMAT modified specimens. The samples were etched in commercially available Kroll’s reagent, which is a dilute solution of nitric and hydrofluoric acids and is the most commonly used etchant for Ti and its alloys to reveal the grain boundaries. Note that the microstructure of the untreated Ti sample (Figure 3a) shows equiaxed grains, a characteristic of a typical undeformed annealed structure. SMAT modified samples (Figure 3b–d), on the other hand, illustrate two distinct regions of (i) deformed grains near the top and bottom surfaces showing deformation twins, and (ii) undeformed or elastically deformed equiaxed grains in the middle. An enlarged view of the same at higher magnification is also shown in Figure 3e. Moreover, the arbitrary limits of the depth of the deformation twinned zone and equiaxed grains are identified as DT and EQ, respectively, in Figure 3.

It may be observed that the depth of the SMAT deformed zone is smaller for the sample treated for 15 min than for that treated for 25 min. This is, however, a qualitative analysis only. A more quantitative measure is provided in terms of hardness values in a later section. Similarly, grain refinement is difficult to be detected from these micrographs. Hence, optical microscopy is deemed insufficient to reveal sufficient information at the nanoscale.

### 3.3. Grain Size Measurement

**Figure 4:**
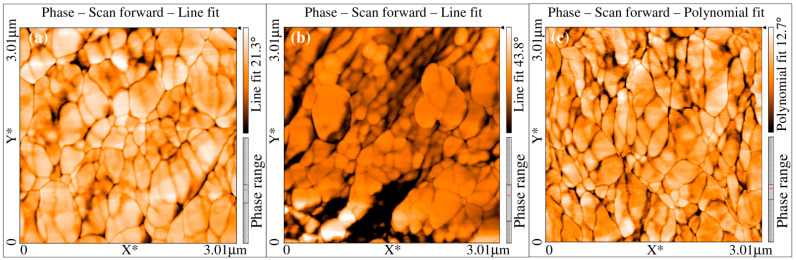
Micrographs of Ti samples showing surface grains of SMAT-modified samples treated for: (**a**) 15 min; (**b**) 20 min and (**c**) 25 min.

The initial grain size of the untreated Ti sample was found in the range from 30 to 70 µm with an average grain size of 50 µm (Figure 3a). However, significant grain refinement was observed in SMAT-modified Ti samples. The grain size of SMAT-deformed samples for 15, 20 and 25 min was found in the range of 100 nm to 250 nm as shown in Figure 4. Note that the dark regions in Figure 4 correspond to the high surface roughness of the samples. In these areas, the AFM cantilever could not approach the material surface and, hence, the dark regions appeared due to the unavailability of the signals.

**Figure 5:**
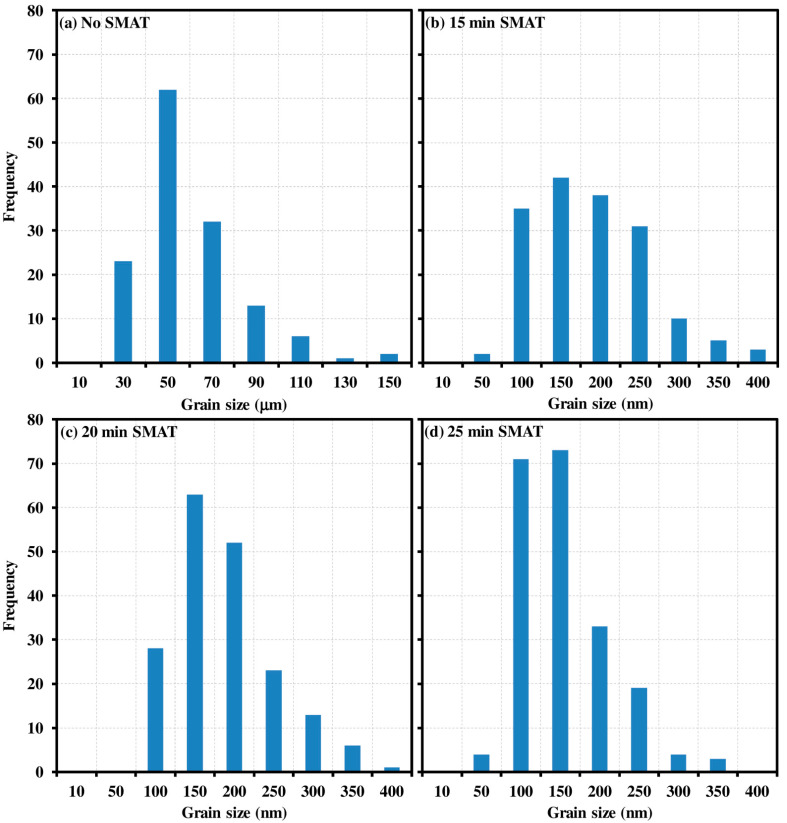
Grain size frequency distribution of: (**a**) untreated Ti; SMAT-modified samples treated for: (**b**) 15 min; (**c**) 20 min and (**d**) 25 min.

Figure 5 shows the grain size distribution of untreated Ti and SMAT deformed samples. It may be observed that the majority of grains fall within the range of 30–70 μm, with a maximum frequency of 50 μm grains for untreated Ti (Figure 5a). Whereas most of the grains of SMAT modified samples lie within 100–250 nm with a maximum number of grains of an average size of 150 nm in each case. However, there seems to be a direct relation between SMAT time and the maximum number of average-sized grains. For instance, 15 min, 20 min and 25 min duration of SMAT yielded more than 40, 60 and 70 numbers of grains of size 150 nm, respectively. In addition, increasing the SMAT time led to a right-skewed frequency distribution, especially in the case of 25 min SMAT where the frequency of 100 nm grains was more than twice (71 grains) in comparison to those of 15 min (35 grains) and 20 min (28 grains) SMAT for similar grain size. Although the numbers of grains scanned in each case were different, the normalized frequency was consistent. For example, the normalized frequencies of 100 nm grains were 0.21 (=35/166), 0.15 (=28/186) and 0.34 (=71/207) for 15 min, 20 min and 25 min SMAT, respectively.

### 3.4. Hardness Profile

**Figure 6:**
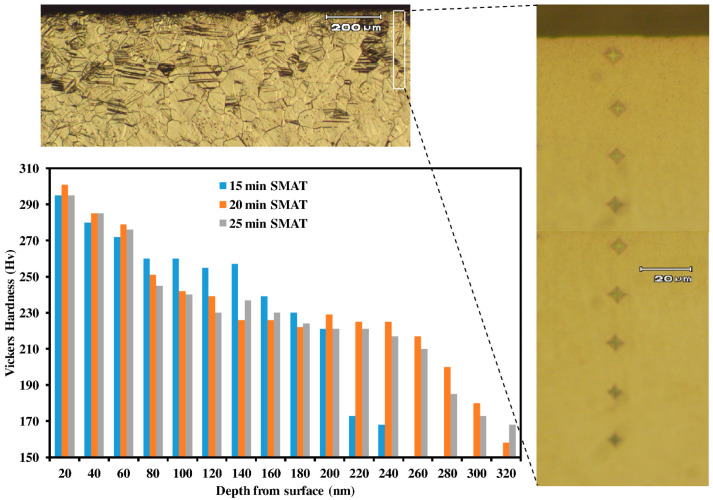
Vickers hardness profile as a function of depth from surface for samples with 15 min, 20 min and 25 min SMAT time.

The Vickers hardness of the untreated Ti with unstressed equiaxed grains was found to be 162 HV on average. Figure 6 shows hardness profiles as a function of the depth of all the SMAT-modified samples, where the Vickers hardness was measured from the top surface to the middle of each specimen at equal intervals of 20 μm. Note that the hardness of the SMAT-modified surface layer (~300 HV) increased by almost 100% as compared to that of the untreated sample. Moreover, it may be noticed that the hardness values remain constant near the surface but decrease gradually in the thickness direction. The depth of the SMAT-modified layer, however, appears to be a function of SMAT time, as an increase in the effective depth may be observed with an increase in the SMAT duration. Obviously, the decrease in hardness could be attributed to the difference in grain size and the presence of deformation twins. Since the surface grains experienced SPD, in addition to having deformation twins, the maximum grain size refinement and, hence, the maximum hardness were recorded here.

### 3.5. Surface Energy Determination

Surface energies of all the samples were determined using Equation (1), where some of the parameters, such as $\gamma_{L}$, $\gamma_{L}^{d}$ and $\gamma_{L}^{p}$, were known from literature and are summarized in Table 1. The unknowns $\gamma_{S}^{d}$ and $\gamma_{S}^{p}$ can be calculated by solving simultaneous equations, provided *θ* values are measured by observing the spread of ethylene glycol and distilled water droplets on the surface of the specimens. An average of five *θ* values was taken as an angle measure for each case and is given in Table 2. In addition, the calculated polar ($\gamma_{S}^{p}$) and dispersive ($\gamma_{S}^{d}$) components of surface energy and the surface energy of solid ($\gamma_{S}=\gamma_{S}^{p}+\gamma_{S}^{d}$) for undeformed and SMAT deformed samples are also reported. Note that increasing SMAT time decreases the contact angle *θ* of both the liquids, while increasing the surface energy of the samples from 36.91 mJ·m^−2^ to 45.99 mJ·m^−2^, i.e., by a factor of 25% approximately. In other words, the surface energy is proportional to the SMAT time because SMAT leads to the formation of nanograins and high grain boundary density at the surface of the specimen. Furthermore, the increase in surface energy results in an increase in attractive/cohesion forces between the liquid drops and the solid surface, which, in turn, promotes the spread of droplets and decreases the contact angle, *θ*.

### 3.6. Mechanical Properties

**Figure 7:**
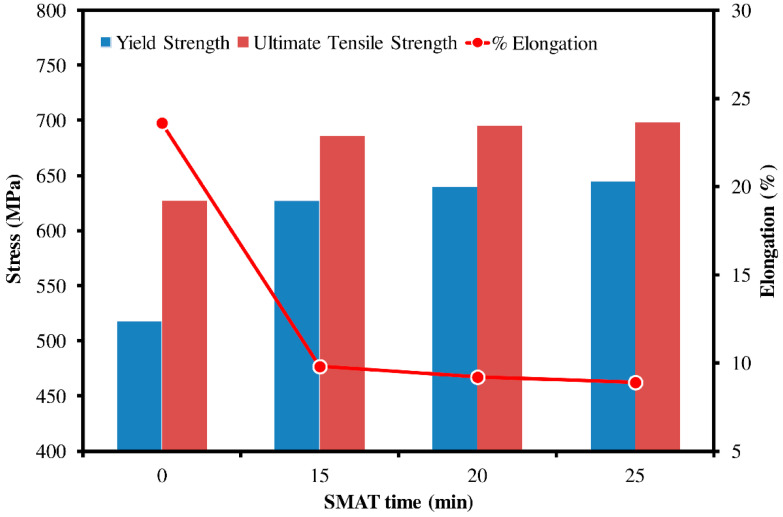
Mechanical properties of Ti samples as a function of SMAT time.

Results of mechanical testing of undeformed and SMAT-deformed Ti samples showed a significant difference in properties since Ti responds readily to work hardening. Figure 7 compares the yield strength, ultimate tensile strength and percentage elongation of all the specimens as a function of SMAT time, where a SMAT time of zero refers to the properties of the undeformed equiaxed Ti sample. As expected, the yield and ultimate strength increased with an increase in SMAT duration by a factor of almost 25% and 10%, respectively. Ductility, or the percentage elongation, however, decreased with increasing SMAT time by a factor of more than 2.5 for similar reasons as explained in the previous sections, such as grain refinement, etc. It has been mentioned earlier that the formation of wear debris is a common failure mode of bio-implants; hence, a low-strength material such as pure Ti is likely to yield and wear out quickly unless the strength and hardness of the interacting surface of the implants are improved. The major advantage of SMAT is that it only improves the surface characteristics up to a certain depth, while the soft core of the material remains intact. Here, the increased strengths and hardness of the surface enhance wear resistance, whereas the soft inner core allows shock resistance.

### 3.7. Corrosion Rate Analysis

**Figure 8:**
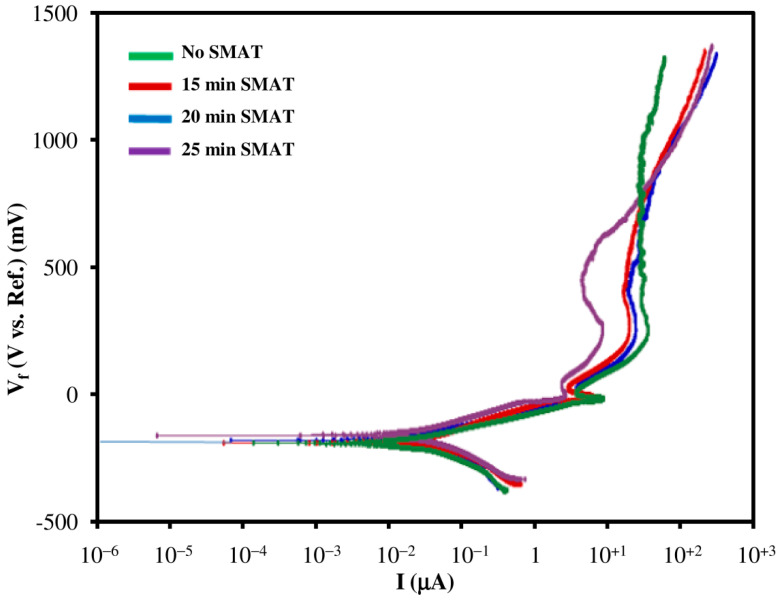
Potentiodynamic polarization curves of undeformed and SMAT deformed samples.

**Figure 9:**
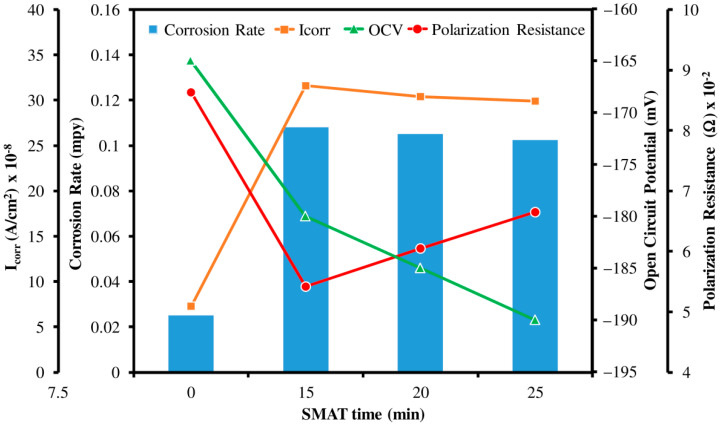
Corrosion rate, Icorr, polarization resistance and open circuit potential as a function of SMAT time.

Potentiodynamic polarization curves of untreated and SMAT modified Ti samples are shown in Figure 8, whereas a comparison of corrosion rates, Icorr, open circuit potential (OCP) and polarization resistance (Rp) is illustrated in Figure 9. Note that the undeformed pure Ti sample has relatively low Icorr and high Rp as compared to SMAT-modified samples. It should be mentioned that low Icorr and high Rp generally correspond to low corrosion rates. On the contrary, SMAT deformed samples show almost identical, yet relatively high corrosion rates. Moreover, the passivation of SMAT samples occurs at lower currents as compared to untreated Ti (see Figure 8). Here, high Icorr and corrosion rates may be attributed to the presence of nanograins at the surface of SMAT deformed samples. This is because a smaller grain size and a high grain boundary density supply an excess of high energy sites at the surface of the material, thereby rendering it more susceptible to corrosion attack.

A sudden increase in Icorr and corrosion rate (Figure 9) for all the SMAT samples provides evidence of the shift of tendency-to-corrode towards anodic behavior. This anodic behavior then leads to the oxidation of Ti and the formation of a passive biocompatible oxide layer around the sample. The high corrosion rate and the low passivation current validate that the passive layer on SMAT-modified Ti samples forms rather quickly. Hence, it may be stated that the presence of SMAT-modified structures, including nanograins and high grain boundary density, would readily form a dense passive film of TiO_2_ at low current values around the sample.

**Figure 10:**
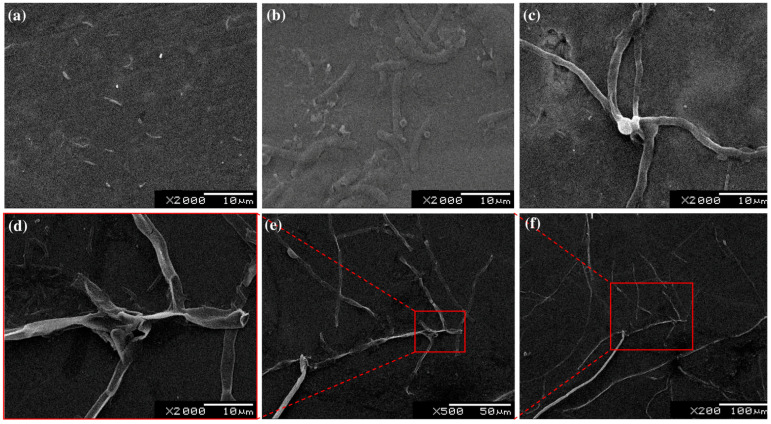
SEM images of: (**a**) untreated Ti; SMAT-modified samples treated for: (**b**) 15 min; (**c**) 20 min and (**d**) 25 min showing deposit of non-toxic salts from ringer lactate solution at reactive grain boundaries; (**e**,**f**) are zoomed-out images of (**d**) showing network of salt deposits in the vicinity.

Figure 10 illustrates the deposit of non-toxic salts on the grain boundaries when untreated and SMAT-modified samples were immersed in Ringer Lactate solution for a period of 360 h. The untreated Ti sample (Figure 10a) shows little to no deposit on its surface, while a gradual increase in salt deposit may be observed as a function of increasing SMAT time (Figure 10b–d). Figure 10e,f highlight an almost continuous network of salt deposits, which validates the increase in hydrophilicity of the Ti sample. Finally, it is important to mention that no alloy system appears free from potential biological hazards. The passive state is relatively unstable and subject to damage under certain conditions, which is beyond the scope of this paper. However, SMAT has no or very little adverse effect on the passivity of Ti.

## 4. Conclusions

In this study, ultrasonic SMAT was applied to the commercially pure Ti to enhance its tribological and mechanical properties along with improving its biocompatibility. Short-duration (15–20 min) SMAT was found to be highly effective since it yielded grain refinement of up to 500 times. Recall that the surface grains (~50 µm) of Ti have transformed into nanograins of a size as small as 100 nm. The presence of nanograins and high grain boundary density was reflected in the form of improved mechanical properties. When compared to the soft Ti core, the hardness of surface layers doubled, the yield strength increased by 25% and the ultimate tensile strength also improved (~10%) somewhat. Another advantage of SMAT was increased surface energy of the Ti samples that, though promoted corrosion rate, assisted an early development of TiO_2_ passive layer at low currents, which, in turn, improved the biocompatibility of Ti. Moreover, an increase in SMAT duration led to an increase in hydrophilicity as observed through the network of salt deposits on the sample surface. Although strengthening improved the wear characteristics of the surface, the associated lowering of ductility reduced toughness and increased brittleness. The SMAT duration is, therefore, a critical parameter that may determine an optimum combination of strength, hardness, ductility, corrosion rate and hydrophilicity.

## Figures and Tables

**Table 1:** Surface energies and polar and dispersive components of distilled water and ethylene glycol [31].

Units (mJ·m^−2^)	Distilled Water	Ethylene Glycol
Surface energy ($\gamma_{L}$)	72.8	48.0
Polar component ($\gamma_{L}^{p}$)	51.0	19.0
Dispersive component ($\gamma_{L}^{d}$)	21.8	29.0

**Table 2:** Surface energy calculation based upon angle *θ* and SMAT time.

SMAT Time (min)	0	15	20	25
*θ*—Distilled water (°)	68.	62.5	57.7	54.6
*θ*—Ethylene glycol (°)	41.2	40.6	39.6	38.5
Polar component (solid)—$\gamma_{S}^{p}$ (mJ·m^−2^)	20.9	14.78	10.99	9.21
Dispersive comp (solid)—$\gamma_{S}^{d}$ (mJ·m^−2^)	16.01	24.38	31.96	36.78
Surface energy—$\gamma_{S}$ (mJ·m^−2^)	36.91	39.16	42.95	45.99

## Data Availability

Not applicable.
